# Efficacy of one dose vaccination against experimental infection with two *Mycoplasma hyopneumoniae* strains

**DOI:** 10.1186/s12917-017-1195-0

**Published:** 2017-08-29

**Authors:** Annelies Michiels, Ioannis Arsenakis, Filip Boyen, Roman Krejci, Freddy Haesebrouck, Dominiek Maes

**Affiliations:** 1 Department of Reproduction, Obstetrics and Herd Health, Faculty of Veterinary Medicine, Ghent University, Merelbeke, Belgium; 2 Department of Pathology, Bacteriology and Avian Diseases, Faculty of Veterinary Medicine, Ghent University, Merelbeke, Belgium; 3 CEVA Santé Animale, Cedex, Libourne, France

**Keywords:** *Mycoplasma hyopneumoniae*, Double challenge, Vaccination

## Abstract

**Background:**

*Mycoplasma hyopneumoniae* (*M. hyopneumoniae*) is the primary agent of enzootic pneumonia in pigs. Pigs are often infected with different *M. hyopneumoniae* strains. This study assessed the efficacy of vaccination against experimental infection with two genetically different *M. hyopneumoniae* strains in weaned piglets. At 33 days of age (D0), 45 *M. hyopneumoniae*-free piglets were randomly assigned to three different groups: 1) negative control group (NCG; *n* = 5): not vaccinated, not infected, 2) positive control group (PCG; *n* = 20): not vaccinated, infected, and 3) vaccination group (VG; *n* = 20): single vaccination with an inactivated whole-cell *M. hyopneumoniae* vaccine (Hyogen®, Ceva) (D1), infected. The PCG and VG were endotracheally inoculated with 7 × 10^7^ CCU in 7 ml of the highly virulent *M. hyopneumoniae* strain F7.2C (D24) and 7 × 10^7^ CCU in 7 ml low virulent strain F1.12A (D25). A respiratory disease score (RDS) was assessed from D24 until D53. At D53 (euthanasia), macroscopic lung lesions (MLL) were scored, log copies of *M. hyopneumoniae* DNA (qPCR) and IL-1 and IL-6-concentrations (ELISA) on bronchoalveolar lavage fluid were determined.

**Results:**

The RDS and MLL at euthanasia were respectively 0, 1.20 and 0.55 (*P* < 0.001) and 0, 7.56 and 0.68 (*P* < 0.001) for NCG, PCG and VG, respectively. The qPCR results for PCG and VG were 3.99 and 1.78 log copies (*P* < 0.001), respectively, with a significant difference between PCG and VG. The IL-1 and IL-6 results at euthanasia for NCG, PCG and VG were 17.61, 1283.39 and 53.04 pg/ml (*P* < 0.001) and 148.10, 493.35 and 259.80 pg/ml (*P* = 0.004), respectively with a significant difference between PCG and VG.

**Conclusions:**

Vaccination with Hyogen® in pigs was efficacious against an experimental challenge with both a low and highly virulent *M. hyopneumoniae* strain as the vaccinated pigs coughed significantly less, and showed significantly less lung lesions compared to the non-vaccinated challenged pigs: the vaccinated animals showed a 52.9% lower RDS and 91.0% lower MLL compared to the PCG. In the bronchoalveolar lavage fluid collected at the necropsy of the vaccinated pigs, a significantly lower amount of *M. hyopneumoniae*-DNA and a significantly lower IL-1 and IL-6 concentration was found compared to the pigs of the PCG.

## Background

*Mycoplasma hyopneumoniae* (*M. hyopneumoniae*) is the causative agent of enzootic pneumonia [1]. The disease has a worldwide impact on intensive swine production and causes substantial losses due to reduced growth of the pigs, poor feed conversion ratio, higher antimicrobial use and increased susceptibility to secondary respiratory agents [1–3]. Previous research has shown that most pigs are infected with more than one *M. hyopneumoniae* strain [4, 5], and recently, Michiels et al., [6] showed that a higher severity and prevalence of *Mycoplasma*-like lung lesions was found in batches of slaughter pigs with detection of more than one *M. hyopneumoniae* strain [6]. It is generally accepted that control of *M. hyopneumoniae* is critical for reducing economical losses in infected swine operations [7]. This is achieved by optimizing housing conditions, management practices, antimicrobial therapy and vaccination against *M. hyopneumoniae* [8]. It is estimated that 70% of swine herds are practising vaccination against *M. hyopneumoniae* worldwide [9]. Vaccination reduces clinical signs, lung lesions and improves performance, although colonization is not prevented and there is no significant reduction in transmission [3, 8, 10–12]. The benefits obtained through vaccination can vary from herd to herd [13]. Most commercially available bacterin vaccines are based on an adjuvanted whole-cell preparation of an inactivated *M. hyopneumoniae* strain [8]. This strain is mostly the J-strain of *M. hyopneumoniae*, which was isolated in 1958 [14, 15]. Recently a commercial bacterin became available, using *M. hyopneumoniae* strain 2940, isolated in the 1999’s from a farm in the United States facing a severe outbreak of enzootic pneumonia. Also, most vaccination studies so far used one single strain for experimental infection. As most pigs are infected with more than one *M. hyopneumoniae* strain [4–6], it may be more appropriate to challenge the pigs with different *M. hyopneumoniae* strains.

The aim of the present study was to determine the efficacy of a commercial vaccine (Hyogen®) against an experimental challenge with two genetically different (low and highly virulent) *M. hyopneumoniae* strains.

## Methods

### Study animals and experimental design

The study was performed after approval by the Ethical Committee for Animal Experiments of the Faculty of Veterinary Medicine, Ghent University (approval number EC2014/165). Forty-five *M. hyopneumoniae*-free Rattrelow-Seghers (RA-SE Genetics NV, Ooigem, Belgium) piglets were enrolled in the study. The herd of origin has been free of *M. hyopneumoniae* for many years based on repeated serological testing, absence of clinical signs and pneumonia lesions, and nPCR testing on tracheobronchial swabs. The piglets were free of following pathogens as well: porcine reproductive and respiratory syndrome virus, *Pasteurella multocida* and *Actinobacillus pleuropneumoniae*. The piglets were weaned at 28 days of age and moved to the experimental facilities of the Faculty of Veterinary Medicine, Ghent University, Belgium. They were housed in experimental chambers with absolute filters (HEPA U15) and were fed ad libitum using a non- antimicrobial supplemented diet. At 33 days of age (D0), the piglets were randomly allocated to three different groups (D0): 1) negative control group (NCG; *n* = 5): not vaccinated, not infected, 2) positive control group (PCG; *n* = 20): not vaccinated, infected, and 3) vaccination group (VG; *n* = 20): single vaccination with a one- dose commercial vaccine (Hyogen®, CEVA Santé Animale, Libourne Cedex, France) (D1), infected.

Hyogen® is a whole-cell bacterin based on strain BA 2940–99, oil adjuvanted with paraffin and *Escherichia coli* J5 LPS with thiomersal as excipient. The animals of the VG were intramuscularly vaccinated with two ml of the commercial vaccine. The animals of the PCG and NCG were injected intramuscularly with two mL of phosphate-buffered saline (PBS).

### *Mycoplasma hyopneumoniae* strains and challenge infection

The highly virulent F7.2C and the low virulent *M. hyopneumoniae* strain F1.12A were used for challenge infection [16]. The pigs were anesthesized with 0.22 ml/kg of a mixture of Zoletil 100® (Virbac, Louvain la Neuve, Belgium) and Xyl-M® 2% (VMD, Arendonk, Belgium). The pigs of the PCG and VG were endotracheally inoculated with 7 × 10^7^ CCU in 7 ml inoculum of strain F7.2C at D24 and with 7 × 10^7^ CCU in 7 ml inoculum of strain F1.12A at D25. Pigs of the NCG were endotracheally inoculated with 7 ml sterile culture medium (Friis medium) at D24 and D25. At four weeks post-inoculation (PI) (D53), all piglets were euthanized. Therefore, deep anaesthesia was applied by intramuscularly administrating 0.3 ml/kg of a mixture of Zoletil 100® (Virbac, Louvain la Neuve, Belgium) and Xyl-M® 2% (VMD, Arendonk, Belgium), followed by exsanguination.

### Clinical and performance parameters

The piglets were observed for at least half an hour daily from D0 until D53. Body condition, appetite, manure consistence, and presence of dyspnea or tachypnea were evaluated. A respiratory disease score (RDS) was recorded daily by the same person at eight a.m. from D1 until D53 and could range from 0 to 6 with 0 (no coughing), 1 (mild coughing after encouraged move), 2 (mild coughing in rest), 3 (moderate coughing after encouraged move), 4 (moderate coughing in rest), 5 (severe coughing after encouraged move), 6 (severe coughing in rest) [17]. The daily RDS values were averaged for the following periods: D1- D23, D24– D53, D1-D53. All pigs were weighed at D0, the day of the first inoculation (D24) and the day of euthanasia (D53), to calculate the anesthesia dose discussed above. The pigs were weighed at D0, D24 and D53 (g). The average daily gain (ADG) (g/pig/d) from day 0–24, 24–53 and from day 0–53 was calculated according to Del Pozo Sacristán et al. (2014), [18] by subtracting the starting weights from the final weights divided by the number of days during the respective periods.

### Macroscopic and histopathologic lung lesions

The lungs were collected and scored for macroscopic lung lesions (MLL) (0–35) according to Hannan et al. (1982) [19]. Samples from the right apical, cardiac and diaphragmatic lung lobe were collected. If lesions were present, the samples were collected from the border of a lesion. Samples were fixated in 10% neutral formalin and processed and embedded in paraffin for histopathological examination. The samples were stained with hematoxylin and eosin and scored using light microscopy according to the degree of peribronchiolar and perivascular lymphohistiocytic infiltration and nodule formation (cuffing) [20]. The scoring system ranged from 1 to 5 with 1) limited infiltration of macrophages and lymphocytes around bronchioles, with airways and alveolar spaces free of cellular exudates; score 2) light to moderate infiltrates with mild diffuse cellular exudates into airways; score 3, score 4 and score 5 (respectively mild (score 3), moderate (score 4) and severe (score 5) lesions characteristic of broncho-interstitial pneumonia, centered around bronchioles but extending to the interstitium, with lymphofollicular infiltration and mixed inflammatory cell exudates. Scores 1 and 2 are considered not to be related with *M. hyopneumoniae* infection, while scores 3 to 5 are presumptive of a *M. hyopneumoniae* infection. The percentage of lung area occupied by air (percentage air) was examined by means of an automated image analysis system (Leica application suite AF Lite (Diegem, Belgium) and image J (Bethesda, Maryland, USA)) [21]. This parameter is inversely proportional to the lymphohistiocytic infiltration in the lung tissue and the intrabronchiolar-and bronchial exudate.

### Quantitative PCR for f *M. hyopneumoniae*

Bronchoalveolar lavage fluid was collected from all animals two weeks PI (D39) by inserting a catheter (Portex® Dog Catheter with Female Luer Mount, Smiths Medical International Ltd. Kent, United Kingdom) in the trachea by means of a mouth gag and whilst snaring the pigs. Next, the lungs were flushed with 20 mL sterile PBS to collect the bronchoalveolar lavage fluid. Additionally, during necropsy (D53), bronchoalveolar fluid was collected from the left part of the lung before collection of the histopathological samples by flushing the head bronchus of the left part of the lung with 20 mL sterile PBS. The bronchoalveolar lavage fluid was divided into seven aliquots and stored at −70 °C awaiting analysis. From one aliquot of the bronchoalveolar lavage samples, DNA was extracted with the QIAGEN protocol (QIAGEN, DNeasy Blood & Tissue kit, Belgium) and quantitative PCR (qPCR) was performed as previously described to detect the numbers of *M. hyopneumoniae* organisms [22]. A tenfold dilution series of *M. hyopneumoniae* DNA of strain F7.2C was used to convert the threshold values to the number of *M. hyopneumoniae* organisms. Values below the dilution of 1.50 × 10^1^ (1.18 log copies) were considered as negative.

### Immunological parameters on bronchoalveolar lavage fluid

#### *M. hyopneumoniae*-specific antibodies in bronchoalveolar lavage fluid

The isotype of *M. hyopneumoniae*-specific antibodies in bronchoalveolar lavage fluid collected at D39 and D53 was determined via an indirect ELISA (expressed in optical density “OD”-values) according to the protocol of Bereiter et al. [23, 24]. Briefly, a Nunc Maxisorp® flat-bottom 96 well plate (eBioscience, Vienna, Austria) was coated with Tween® 20 extracted *M. hyopneumoniae* antigens on which the bronchoalveolar lavage fluid (D39, D53) was added, undiluted. Peroxidase labeled goat anti-porcine Ig A or Ig G polyclonal antibodies (Bethyl Laboratories, Texas, TX, USA) were added and the OD at 450 nm was measured.

#### Cytokines in bronchoalveolar lavage fluid

The bronchoalveolar lavage fluid collected at D39 and D53 was tested undiluted for presence of porcine TNF-α (TNF-α Swine Antibody Pair, Invitrogen), IL-1 (Porcine IL-1 beta/IL-1F2 DuoSet, R&D Systems) and IL-6 (Porcine IL-6 DuoSet, R&D Systems). A sandwich ELISA was performed according to the manufacturers’ recommendations. The sample reactions were measured using OD at 450 nm and quantified by the use of a standard curve as described in the manual.

### Serology

At D1, D24 and at euthanasia (D53), blood was collected from all pigs and tested for the presence of antibodies against *M. hyopneumoniae* with a blocking ELISA (IDEIA™ *Mycoplasma hyopneumoniae* EIA kit, Oxoid Limited, Hampshire, UK), according to the protocol manual. Sera with optical density < 50% of the average value of the OD-buffercontrol were considered to be positive. All values above or equal to 50% of the average value of the OD-buffercontrol were classified as negative.

### Routine bacteriological culture on bronchoalveolar lavage fluid

For the bacteriological examination, ten μL of bronchoalveolar lavage fluid collected on D53 of each pig was inoculated on Columbia agar supplemented with 5% sheep blood (Oxoid, Hampshire, UK) with a *Staphylococcus pseudintermedius* streak [25]*.* Plates were incubated overnight in a 5% CO_2_-enriched environment at 35 °C for 48 h for identification of respiratory bacteria in the lungs.

#### Statistical analysis

Descriptive statistics were performed in order to check the normality of the data. One-way analysis of variance (ANOVA) was used to analyse weight and ADG. Repeated measurements ANOVA was performed to analyze the RDS data. Scheffé’s post hoc test was used to make pairwise group comparisons. The parameters MLL, histopathological lesions, percentage air analysis, ELISA *M. hyopneumoniae*, TNF-α, IL-1, IL-6, Ig A, Ig G and qPCR results were analyzed using a non-parametric Kruskal-Wallis, as the data did not fulfil the assumptions of normality. Theses analyses were performed with SPSS 22 for Windows (SPSS inc. Illinois, USA). Percentage of seropositive pigs and percentage of pigs testing positive with qPCR in each group were analyzed using binomial logistic regression (R version 3.3.1) [26]. Results were considered to be statistically significant when *P* < 0.05.

## Results

No animals died during or shortly after the inoculations. One out of 45 piglets in the NCG was euthanized (D22) (Release®300 mg/ml, WDT, Garbsen, Germany) due to nervous symptoms and lateral decubitus. Necropsy was performed and *Haemophilus parasuis* was isolated in pure culture from the meninges and pericardium. All animals in the PCG coughed, showed macroscopic lung lesions and seroconverted. *Mycoplasma hyopneumoniae*-DNA was detected in 19 out of 20 animals from this group.

### Clinical and performance parameters

**Fig. 1:**
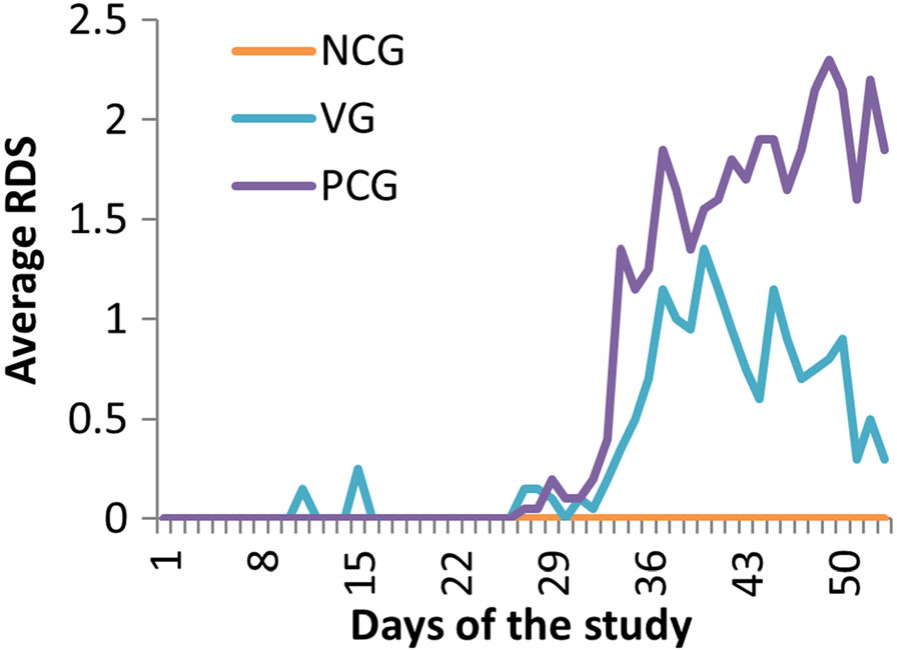
Average respiratory disease score (RDS) during the trial for the negative control group (NCG), positive control group (PCG) and vaccination group (VG). The average RDS was assessed daily from day 1 until day 53. The piglets were vaccinated at D1 and the inoculations were performed at D24 and D25

The results of the clinical parameters are summarized in Table 1. There was no coughing in the NCG throughout the study. The RDS_24–53_ was 1.20 ± 0.83 and 0.55 ± 0.42 for the PCG and VG (*P* < 0.001), respectively. This corresponds with a reduction of 52.9% in RDS when vaccinating the piglets compared to the PCG. All groups significantly differed from each other. These results and the RDS _1–53_ and RDS _1–23_ results are shown in Table 1 and Fig. 1.

**Table 1:** Results of different parameters in the different experimental groups

Parameter	Experimental Groups			
	NCG (# = 5)	PCG (# = 20)	VG (# = 20)	*P*-value
RDS				
D1–53	0 ± 0 ^a^	0.68 ± 0.41^b^	0.32 ± 0.86 ^c^	*<0.001*
D1–23	0 ± 0 ^a^	0 ± 0 ^a^	0.017 ± 0.060 ^a^	0.983
D24–53	0 ± 0 ^a^	1.20 ± 0.83 ^b^	0.55 ± 0.42 ^c^	*<0.001*
Weight ± SD				
D0	7.04 ± 1.22^a^	6.97 ± 0.84 ^a^	7.04 ± 1.02 ^a^	0.966
D24	15.25 ± 3.96 ^a^	16.39 ± 2.16 ^a^	15.61 ± 2.96 ^a^	0.581
D53	36.51 ± 8.63 ^a^	34.63 ± 6.63 ^a^	34.27 ± 5.35 ^a^	0.808
ADG (g/pig/d)				
D0–24	357 ± 123 ^a^	393 ± 62.8 ^a^	357 ± 92.0 ^a^	0.372
D24–53	733 ± 194 ^a^	629 ± 199 ^a^	643 ± 102 ^a^	0.504
D0–53	563 ± 144 ^a^	522 ± 121 ^a^	514 ± 87.0 ^a^	0.714
MLL, histopathology and air analysis D53				
MLL	0 ± 0 ^a^	7.56 ± 4.73 ^b^	0.68 ± 0.73 ^a^	*<0.001*
Histopathology	1.31 ± 0.18 ^a^	3.32 ± 0.85 ^b^	1.92 ± 0.47 ^c^	*<0.001*
Percentage of air (%)	47.72 ± 7.29 ^a^	34.49 ± 8.22 ^b^	45.23 ± 5.99 ^a^	*<0.001*
log copies of *M. hyopneumoniae* DNA/ml BALF (qPCR) ± SD				
D39	0.36 ± 0.64 ^a^	1.81 ± 1.32 ^a^	1.23 ± 1.61 ^a^	0.062
D53	0.40 ± 0.43 ^a^	3.99 ± 1.20 ^b^	1.78 ± 1.36 ^a^	*<0.001*
Percentage of *M. hyopneumoniae* qPCR positive samples in BALF (#positive samples/total number of samples)				
D39	(0/4) 0^a^	(14/20) 70^b^	(7/20) 35^c^	*<0.01*
D53	(0/4) 0^a^	(19/20) 95^b^	(12/20) 60^c^	*<0.001*
*M. hyopneumoniae* specific AB expressed in OD-values ± SD in serum				
D1	1.51 ± 0.037 ^a^	1.48 ± 0.058 ^a^	1.48 ± 0.16 ^a^	0.578
D24	0.91 ± 0.044 ^a^	0.95 ± 0.069 ^a^	0.50 ± 0.17 ^b^	*<0.001*
D53	0.90 ± 0.10 ^a^	0.23 ± 0.11 ^a^	0.087 ± 0.041 ^b^	*<0.001*
Percentage of ELISA *M. hyopneumoniae* positive samples in serum				
D1	(0/5) 0^a^	(0/20) 0^a^	(0/20) 0^a^	1.000
D24	(0/4) 0^a^	(0/20) 0^a^	(15/20) 75^b^	*<0.001*
D53	(0/4) 0^a^	(20/20) 100^b^	(20/20)100^b^	*<0.001*

The respiratory disease score (RDS), body weight, average daily gain (ADG), *M. hyopneumoniae* specific antibodies, macroscopical lung lesions (MLL), histopathology and air analysis, log copies of *M. hyopneumoniae* DNA (qPCR), percentage of of *M. hyopneumoniae* qPCR positive samples, *M. hyopneumoniae* specific AB expressed in OD-values and Percentage of ELISA *M. hyopneumoniae* positive samples

^a,b,c^Different superscripts in one row are statistically different (*P* < 0.05)

*NCG* negative control group, *PCG* positive control group, *VG* vaccination group, *SD* standard deviation, *D* Day of the study, *ADG* average daily gain, *kg* kilogram, *RDS* respiratory disease score, *M. hyopneumoniae Mycoplasma hyopneumoniae, AB* antibodies, *OD* optical densities, *#* number, *MLL* macroscopic lung lesions, *qPCR* quantitative polymerase chain reaction, *DNA* DeoxyriboNucleic Acid, *BALF* bronchoalveolar lavage fluid

The average group weight at D0, D24 and D53 and the ADG calculated during these three periods (0–24, 24–53 and 0–53 days of age) for each group are shown in Table 1.

### Macroscopic and histopathologic lung lesions

There were no macroscopic lung lesions in the NCG. The MLL of the PCG and VG was 7.56 ± 4.73 and 0.68 ± 0.73, respectively (*P* < 0.001) (Table 1). A reduction of 91.0% in MLL of the VG was observed compared to the PCG. The histopathological lung lesion scoring in the NCG, PCG and VG was 1.31 ± 0.18, 3.32 ± 0.85 and 1.92 ± 0.47 (*P* < 0.001), respectively. The percentage of lung area occupied by air (percentage air) was 47.72 ± 7.29, 34.49 ± 8.22 and 45.23 ± 5.99% for the NCG, PCG and VG (*P* < 0.001), respectively. For the MLL and percentage of air analysis, the PCG was significantly different from the VG and NCG. For the histopathology score, all groups significantly differed from each other (Table 1).

### Quantitative PCR for detection of *M. hyopneumoniae* DNA

The samples of the NCG remained negative throughout the study. The number of log copies of *M. hyopneumoniae* detected in the bronchoalveolar lavage fluid with qPCR in the PCG and VG were: 3.99 ± 1.20 (PCG) and 1.78 ± 1.36 (VG) (*P* < 0.001) at D53 (Table 1). The results of D39 and the percentage of animals that tested positive by qPCR are also shown in Table 1.

### Immunological parameters on bronchoalveolar lavage fluid

All results of the immunological parameters on D39 and D53 are summarized in Table 2.

**Table 2:** Immunological parameters measured in the BALF at D39 and D53

Parameter	Experimental groups			
	NCG (# = 5)	PCG (# = 20)	VG (# = 20)	*P*-value
Ig G ± SD (OD-values)				
D39	0 ± 0 ^a^	0.069 ± 0.14 ^a^	0.31 ± 0.65 ^a^	0.366
D53	0.38 ± 0.17 ^a^	2.25 ± 0.91 ^b^	2.41 ± 0.88 ^b^	*0.005*
Ig A ± SD (OD-values)				
D39	0 ± 0 ^a^	0.067 ± 0.18 ^a^	0.55 ± 0.99 ^a^	0.359
D53	0.18 ± 0.14 ^a^	2.10 ± 0.75 ^b^	2.08 ± 0.58 ^b^	*0.005*
TNF-α ± SD (pg/ml)				
D39	23.61 ± 1.74 ^a^	18.56 ± 4.40 ^a^	21.12 ± 4.56^a^	*0.029*
D53	16.03 ± 1.10 ^a^	14.53 ± 5.95 ^a^	15.15 ± 2.40^a^	0.065
IL-1 ± SD (pg/ml)				
D39	32.47 ± 1.77 ^a^	239.63 ± 511.35 ^a^	247.13 ± 606.71^a^	0.367
D53	17.61 ± 1.98 ^a^	1283.39 ± 1075.65 ^b^	53.04 ± 54.23 ^a^	*<0.001*
IL-6 ± SD (pg/ml)				
D39	130.33 ± 18.65 ^ab^	242.24 ± 122.23 ^a^	164.64 ± 84.48 ^b^	0.03
D53	148.10 ± 8.89 ^a^	493.35 ± 494.70 ^b^	259.80 ± 333.38 ^a^	*0.004*

D39: two weeks after challenge

D53: necropsy

^a,b^Different superscripts in one row are statistically different (*P* < 0.05)

*NCG* negative control group, *PCG* positive control group, *VG* vaccination group, *SD* standard deviation, *D* Day of the study, *OD* optical densities, *#* number

#### Detection of *M. hyopneumoniae*-specific antibodies in bronchoalveolar lavage fluid

There were no significant differences in the Ig G and Ig A results between the three groups at D39 (Table 2).

At D53, the Ig G values were 0.38 ± 0.17, 2.25 ± 0.91 and 2.41 ± 0.88 for the NCG, PCG and VG, respectively (*P* = 0.005). At D53, the results for Ig A were 0.18 ± 0.14, 2.10 ± 0.75, 2.08 ± 0.58 for the NCG, PCG and VG, respectively (*P* = 0.005). Both the Ig G and Ig A concentrations at D53 of the PCG and VG differed significantly from the NCG.

#### Detection of cytokines in bronchoalveolar lavage fluid

The TNF-α, IL-1 and IL-6 results at D39 and D53 are shown in Table 2. There were no significant differences obtained in the TNF-α- results (pg/ml) at D39, nor at D53.

At D53, the IL-1 concentration in the bronchoalveolar lavage fluid was 17.61 ± 1.98, 1283.39 ± 1075.65 and 53.04 ± 54.23 pg/ml in the NCG, PCG and VG, respectively (*P* < 0.001).

At D39, the IL-6 concentration was significantly different between the PCG and VG (Table 2). At D53, the IL-6 concentration was 148.10 ± 8.89, 493.35 ± 494.70 and 259.80 ± 333.38 for the NCG, PCG and VG, respectively (*P* = 0.004) with significantly higher concentrations in the PCG compared to the NCG and VG.

### Serology

The serological results are presented in Table 1. All pigs of the NCG remained serologically negative throughout the study at D53 all pigs of the PCG and VG were serologically positive for *M. hyopneumoniae*. The OD values at D53 were 0.90 ± 0.10, 0.23 ± 0.11 and 0.087 ± 0.041 in the NCG, PCG and VG, respectively (*P* < 0.001). In the VG significantly lower OD-values were detected, thus higher *M. hyopneumoniae* specific antibodies compared to the PCG and NCG (Table 1).

### Bacteriological culture

*Streptococcus suis* was isolated in only one pig of the PCG. No relevant bacterial growth was observed in all other lung samples.

## Discussion

This study showed that the vaccine was efficacious for the most part against an experimental challenge with both a low and highly virulent *M. hyopneumoniae* strain. The vaccinated pigs coughed significantly less, showed significantly less lung lesions, a significantly lower number of log copies in the bronchoalveolar fluid at euthanasia was shown and the IL-1 and IL-6 concentrations at euthanasia were lower compared to the non-vaccinated inoculated pigs.

The challenge infection was performed with two genetically different [27] *M. hyopneumoniae* isolates. The virulence of these strains was evaluated by Vicca et al. [16] and both strains were characterised at proteomic and genomic level by Calus et al. [28], Vranckx et al. [4] and Stakenborg et al. [29], respectively. The double challenge infection model was successful as all animals in the PCG showed lung lesions, coughed and seroconverted. In 19 out of 20 animals of the PCG at euthanasia, DNA of *M. hyopneumoniae* was detected in the bronchoalveolar lavage fluid. It is well known that there can be quite some variation between pigs in terms of responses to an experimental infection. Why this particular pig tested negative is not known. Possibly, the conditions for multiplication of the *M. hyopneumoniae* strains were not optimal in that pig. Although bronchoalveolar fluid is an appropriate sampling technique to detect *M. hyopneumoniae* during the early stages of infection, it fails to recover some positive animals in case of chronic infection [30]. Taken these factors into consideration, this might be the reason why, although the pig was challenged, it did not test positive. Although comparing to other experimental settings should be done with caution, clinical symptoms and lung lesions were comparable or more severe than the single F7.2C challenge model performed in our research group [12, 16, 24, 25, 31–38] and the standard deviation was lower [16, 31–35]. It was shown that most pigs in the field are simultaneously infected with two or sometimes three genetically different *M. hyopneumoniae* strains and that when batches of slaughter pigs are infected with more than one *M. hyopneumoniae* strain, this can result in more (severe) pneumonia lesions and fissures [6]. The current experimental setting might therefore better resemble the field situation than infection with a single strain. Finally, it was shown that anesthetizing and inoculating the piglets with seven ml of inoculum on two consecutive days is feasible, as none of the piglets showed adverse reactions or died during or shortly after the inoculations.

The pigs in the vaccinated group coughed significantly less (−53%) from inoculation until euthanasia compared to the positive control group, demonstrating the efficacy of the vaccine. The degree of coughing is an important efficacy parameter [39, 40] not only under experimental but also under field conditions. A coughing index can be used to estimate if a higher prevalence of enzootic pneumonia in a herd might be present [41] and to determine the most optimal timing of serological sampling of the pigs or collecting bronchoalveolar fluid to be tested with PCR [41, 42]. In this study, no statistical significant differences were observed in the parameters weight and ADG, although the pigs in the VG grew slightly faster compared to the PCG from day of inoculation until day of euthanasia onwards. Although the parameter weight and ADG are of great use to evaluate in field circumstances, in experimental trials they are merely of descriptive value as the low number of animals used, the duration of the trial and the sometimes high standard deviations are the reason why only numerical differences are found [10, 16, 31].

The MLL score in the vaccinated pigs was very low (0.68) and statistically different from the PCG (7.56), implying a reduction with 91%, and demonstrating the efficacy of the vaccine. A recent field study showed that when pigs are infected with more strains, more lung lesions are detected [6]. When comparing the MLL scores from the double inoculated challenge in PCG with those from challenged unvaccinated pigs in the single inoculation model from previous studies (highest MLL was 6.69 for F7.2C challenge in Villarreal et al., [25]), higher MLL in this study are obtained. However, comparing different experimental settings, should be done with caution. To make a true comparison, the resulting MLL from the double infection model should be compared with the single inoculation model from both strains F7.2C and F1.12A in one experimental setting.

In the vaccinated animals significantly less lymphohistiocytic infiltration (1.92) was observed compared to the PCG (3.32). These results are in accordance with the results of previous studies [25, 31, 32]. Although many aspects of the effect of vaccination on the immunological response remain unclear [43], the present results confirm that vaccination has a regulating function on the immune system, causing less infiltration of macrophages in the lung tissue [34]. A significantly lower area occupied by air or air percentage was measured in the non-vaccinated animals of PCG (34.49%) compared to VG (45.23%). Infection with *M. hyopneumoniae* results in intrabronchiolar- and bronchial exudate and infiltration of lymphohistiocytic cells in the lung tissue. These responses may result in compressing the airways and alveoli, leading to less air volume in the lungs [15]. Vaccination also significantly reduced the log copies of *M. hyopneumoniae* DNA in the bronchoalveolar lavage fluid compared to the non-vaccinated animals (1.78 versus 3.99), which may lead to less shedding of *M. hyopneumoniae* in vaccinated pigs. Similar reductions were found in previous studies [34, 44]. These results, although very promising, confirm once more that vaccination is not able to prevent pigs from being colonized as commonly known [8].

*Mycoplasma hyopneumoniae*-specific antibody (Ig G and Ig A) levels in tracheo-bronchial washings were not significantly different between vaccinated and non-vaccinated pigs. Our findings are in accordance with Djordevic et al. (1997) [24, 45], but Thacker et al. (2000) suggested that secretion of *M. hyopneumoniae*- antibodies induced by vaccination into the bronchoalveolar washings might be implied in resolving mycoplasmal pneumonia [43, 46, 47]. Further research is needed to clarify a possible protective role of *M. hyopneumoniae*-specific antibodies in trachea-bronchial washings.

There were no significant differences between the vaccinated and non-vaccinated animals for the TNF-α concentration in the bronchoalveolar fluids. In the study of Marchioro et al. [24], vaccination significantly decreased TNF-α concentrations, but the difference was small and only borderline significant. This highlights the need to further elucidate the pathogenesis of *M. hyopneumoniae* and the exact mechanisms in which way *M. hyopneumoniae*-bacterins provide protection to the animal.

The cytokines IL-1 and IL-6, next to TNF-α are pro-inflammatory cytokines [48]. Previous research stated that they mediate lymphocyte infiltration and activation in the pneumonic lung [49, 50], and thus are associated with the induction of pneumonia lesions [24, 51, 52]. The pro-inflammatory cytokines TNF-α, IL-1 and IL-6 were correlated with MLL at D39 and D53. The parameter TNF-α was weakly and not significantly correlated with MLL on both sampling days (D39 and D53). Interleukin 1 was strongly and significantly correlated with MLL at euthanasia, and IL-6 was moderately and significantly correlated with MLL on D39 and D53. This suggests that the vaccine shows a protective effect mainly by modulation of the pro-inflammatory cytokines IL-1 and IL-6, but not TNF-α (data not shown). The IL-1 concentration and the standard deviation in the non- vaccinated pigs was very high. This result is in accordance with the result of Meyns et al. [38] at 28 days post inoculation with the F7.2C isolate. One can conclude that a part of the piglets in the non- vaccinated group showed very high IL-1 levels. The reason why this occurred is not clear, however the individual susceptibility of the pig to an *M. hyopneumoniae* infection must be kept into account causing the challenge infection to be more successful. The IL-1 and IL-6 concentrations at necropsy in the vaccinated pigs were significantly lower than in the non-vaccinated pigs and not significantly different from the levels in negative control group. These results substantiate the statement that was made in Marchioro et al. [24] based on the IL-1 result of the vaccinated pigs at D36 that IL-1 and IL-6 may have a regulatory role upon *M. hyopneumoniae* vaccination [24].

In this study, the pigs were vaccinated approximately 4 weeks before challenge, as vaccination is most likely to be effective if active immunity is established before the piglets are exposed to the pathogen. Under field conditions, however, the time between vaccination and infection is not known, and very likely highly variable between pigs. Hence, it is possible that the vaccine used in the field is less efficacious when the timing of vaccination is not optimal. As this is an experimental study, the results cannot be extrapolated as such to field conditions. Under field circumstances, more factors challenging the efficacy of a vaccine are present, compared to the controlled environment of an experimental facility. First, multi-factorial and multi-pathogen enzootic pneumonia outbreaks are typically found in the field, rather than isolated *M. hyopneumoniae* outbreaks [2]. The piglets originated from a herd free of important diseases which can influence the outcome of an *M. hyopneumonia*e infection, such as PRRSv *Pasteurella multocida*, and *Actinobacillus pleuropneumoniae.* [1, 53, 54] and according to some authors infection with certain pathogens can determine the effectiveness of *M. hyopneumoniae* vaccination as well [54–56]. Second, the impact of weaning stress should be taken into account when vaccinating piglets at weaning age in the field. Although the impact of weaning stress on vaccine efficacy might not be clear [31], it could not have influenced the efficacy of the vaccine, as the pigs were transported and vaccinated some days after weaning. Thirdly, passively acquired maternal derived immunity could not have influenced the efficacy of the vaccine as these piglets were originating from a herd free of *M. hyopneumoniae* and no vaccinations against *M. hyopneumoniae* in the sows, nor the piglets were performed. This is a double sided given. On the one hand, Hodgins et al. [57] stated that maternal antibodies in the piglets were associated with reduced antibody responses to vaccination. On the other hand, the piglets in the study could not benefit from the passively transferred cell-mediated immunity. Bandrick et al. [30] showed that vaccination of piglets against *M. hyopneumoniae* in the face of antigen-specific maternal-derived immunity results in cell-mediated immunity priming and anamnestic cell-mediated immunity responses following the exposure *to M. hyopneumoniae* antigen. On the other hand, some factors in the experimental study were comparable to field circumstances or challenged the vaccine more. First, although the piglets were free from certain pathogens, other than that, the piglets were originating from a conventional farm. Second: the piglets were transported from the herd of origin to the experimental facilities and the piglets needed to establish a new hierarchy after comingling. These events are associated with stress, which might influence the efficacy of the vaccine. This can resemble the stress that piglets experience in the field, when sorted and moved from one facility to another in a multi-site production system. Thirdly: the vaccine was highly challenged in this experimental study, because as stated by Villarreal et al., [35] and Meyns et al. [37], it can be assumed that the *M. hyopneumoniae* challenge dose used to infect the pigs was higher than might be reached under natural conditions resulting in a faster and higher colonization level and presumably challenging the vaccine more than under field circumstances [35, 37]. Although extrapolation of the efficacy testing of the vaccine results obtained in experimentally *M. hyopneumoniae* infected pigs should be done with caution, this infection model enabled to study the effect of the vaccine in *M. hyopneumoniae* infected pigs in a reproducible and standardized way.

## Conclusion

Using a double challenge infection model with a low and highly virulent *M. hyopneumoniae* strain, one-dose vaccination of piglets was efficacious for the most part, as coughing was reduced by more than 50% and macroscopical lung lesions by 91%. In addition, the lymphohistiocytic infiltration in lung tissue was lower, the number of log copies of *M. hyopneumoniae* DNA detected in bronchoalveolar lavage fluid and the IL-1 and IL-6 concentrations at euthanasia were lower in the vaccinated animals compared to non-vaccinated animals.

## Abbreviations

#: Number
ADG: Average daily gain
ANOVA: Analysis of variance
BALF: Bronchoalveolar lavage fluid
D: Day of the study
DNA: DeoxyriboNucleic Acid
*M. hyopneumoniae*: *Mycoplasma hyopneumoniae*
MLL: Macroscopic lung lesions
NCG: Negative control group
OD: Optical density
PBS: Phosphate-buffered saline
PCG: Positive control group
PI: Post-inoculation
qPCR: Quantitative polymerase chain reaction
RA-SE genetics: Rattrelow-Seghers genetics
RDS: Respiratory disease score
SD: Standard deviation
VG: Vaccination group

## References

[1] Maes D, Segales J, Meyns T, Sibila M, Pieters M, Haesebrouck F (2008). Control of *Mycoplasma hyopneumoniae* infections in pigs. Vet Microbiol.

[2] Maes D, Deluyker H, Verdonck M, Castryck F, Miry C, Vrijens B (1999). Effect of vaccination against *Mycoplasma hyopneumoniae* in pig herds with an all-in/all-out production system. Vaccine.

[3] Villarreal I, Meyns T, Dewulf J, Vranckx K, Calus D, Pasmans F (2011). The effect of vaccination on the transmission of Mycoplasma hyopneumoniae in pigs under field conditions. Vet J.

[4] Vranckx K, Maes D, Calus D, Villarreal I, Pasmans F, Haesebrouck F (2011). Multiple locus variable number of tandem repeats analysis is a suitable tool for the differentiation of *Mycoplasma hyopneumoniae* strains without cultivation. J Clin Microbiol.

[5] Vranckx K, Maes D, Del Pozo SR, Pasmans F, Haesebrouck F (2011). A longitudinal study of the diversity and dynamics of *Mycoplasma hyopneumoniae* infections in pig herds. Vet Microbiol.

[6] Michiels A, Vranckx K, Piepers S, Del Pozo SR, Arsenakis I, Boyen F (2017). Impact of diversity of *Mycoplasma hyopneumoniae* strains on lung lesions in slaughter pigs. Vet Res.

[7] Pointon AMD, Byrt D, Heap P (1985). Effect of enzootic pneumonia of pigs on growth performance. Aust Vet J.

[8] Haesebrouck F, Pasmans F, Chiers K, Maes D, Ducatelle R, Decostere A (2004). Efficacy of vaccines against bacterial diseases in swine: what can we expect?. Vet Microbiol.

[9] Martelli P, Saleri R, Cavalli V, De Angelis E, Ferrari L, Benetti M (2014). Systemic and local immune response in pigs intradermally and intramuscularly injected with inactivated *Mycoplasma hyopneumoniae* vaccines. Vet Microbiol.

[10] Jensen CS, Ersbøll AK, Nielsen JP (2002). A meta-analysis comparing the effect of vaccines against *Mycoplasma hyopneumoniae* on daily weight gain in pigs. Prev Vet Med.

[11] Kyriakis SC, Alexopoulos C, Vlemmas J, Sarris K, Lekkas S, Koutsoviti-Papadopoulou M (2001). Field study on the efficacy of two different vaccination schedules with HYORESP® in a *Mycoplasma hyopneumoniae*-infected commercial pig unit. J Vet Med.

[12] Meyns T, Dewulf J, de Kruif A, Calus D, Haesebrouck F, Maes D (2006). Comparison of transmission of *Mycoplasma hyopneumoniae* in vaccinated and non-vaccinated populations. Vaccine.

[13] Martelli P, Terreni M, Guazzetti S, Cavirani S (2006). Antibody response to *Mycoplasma hyopneumoniae* infection in vaccinated pigs with or without maternal antibodies induced by sow vaccination. J Vet Med.

[14] Goodwin RFW, Whittlestone P (1963). Production of enzootic pneumonia in pigs with an agent grown in tissue culture from the natural disease. Br J Exp Pathol.

[15] Marchioro SB, Maes D, Haesebrouck F, Dellagostin OA. Immune responses following vaccination of pigs and mice against *Mycoplasma hyopneumoniae*. Ghent: Ghent Univeristy; 2013.

[16] Vicca J, Stakenborg T, Maes D, Butaye P, Peeters J, de Kruif A (2003). Evaluation of virulence of *Mycoplasma hyopneumoniae* field isolates. Vet Microbiol.

[17] Halbur PG, Paul PS, Meng XJ, Lum MA, Andrews JJ, Rathje JA (1996). Comparative pathogenicity of nine US porcine reproductive and respiratory syndrome virus (PRRSV) isolates in a five-week-old cesarean-derived, colostrum-deprived pig model. J Vet Diagn Investig.

[18] Del Pozo SR, Sierens A, Marchioro SB, Vangroenweghe F, Jourquin J, Labarque G, et al. Efficacy of early *Mycoplasma hyopneumoniae* vaccination against mixed respiratory disease in older fattening pigs. Vet Rec. 2014;174 10.1136/vr.101597 24436349

[19] Hannan PC, Bhogal BS, Fish JP (1982). Tylosin tartrate and tiamutilin effects on experimental piglet pneumonia induced with pneumonic pig lung homogenate containing mycoplasmas, bacteria and viruses. Res Vet Sci.

[20] Morris C, Gardner I, Hietala S, Carpenter T, Anderson R, Parker K (1995). Seroepidemiologic study of natural transmission of *Mycoplasma hyopneumoniae* in a swine herd. Prev Vet Med.

[21] Rasband WS. ImageJ U. S. National Institutes of Health, Bethesda. 1997–2016. https://imagej.nih.gov/ij/. Accessed 17 Aug 2017.

[22] Marois C, Dory D, Fablet C, Madec F, Kobisch M (2010). Development of a quantitative real-time TaqMan PCR assay for determination of the minimal dose of *Mycoplasma hyopneumoniae* strain 116 required to induce pneumonia in SPF pigs. J Appl Microbiol.

[23] Bereiter M, Young TF, Joo HS, Ross RF (1990). Evaluation of the ELISA and comparison to the complement-fixation test and radial immunodiffusion enzyme assay for detection of antibodies against *Mycoplasma hyopneumoniae* in swine serum. Vet Microbiol.

[24] Marchioro SB, Maes D, Flahou B, Pasmans F, Del Pozo SR, Vranckx K (2013). Local and systemic immune responses in pigs intramuscularly injected with an inactivated *Mycoplasma hyopneumoniae* vaccine. Vaccine.

[25] Villarreal I, Maes D, Vranckx K, Calus D, Pasmans F, Haesebrouck F (2011). Effect of vaccination of pigs against experimental infection with high and low virulence *Mycoplasma hyopneumoniae* strains. Vaccine.

[26] Team RC. R: A language and environment for statistical computing. Vienna, Austria. 2016. URL https://www.R-project.org/. Accessed 17 Aug 2017.

[27] Stakenborg T, Vicca J, Butaye P, Maes D, Peeters J, de Kruif A (2005). The diversity of *Mycoplasma hyopneumoniae* within and between herds using pulsed-field gel electrophoresis. Vet Microbiol.

[28] Calus D, Baele M, Meyns T, de Kruif A, Butaye P, Decostere A (2007). Protein variability among *Mycoplasma hyopneumoniae* isolates. Vet Microbiol.

[29] Stakenborg T, Vicca J, Maes D, Peeters J, de Kruif A, Haesebrouck F (2006). Comparison of molecular techniques for the typing of *Mycoplasma hyopneumoniae* isolates. J Microbiol Methods.

[30] Moorkamp L, Nathues H, Spergser J, Tegeler R, Grosse Beilage E (2008). Detection of respiratory pathogens in porcine lung tissue and lavage fluid. Vet J.

[31] Arsenakis I, Panzavolta L, Michiels A, Del Pozo SR, Boyen F, Haesebrouck F (2016). Efficacy of *Mycoplasma hyopneumoniae* vaccination before and at weaning against experimental challenge infection in pigs. BMC Vet Res.

[32] Marchioro SB, Del Pozo SR, Michiels A, Haesebrouck F, Conceição FR, Dellagostin OA (2014). Immune responses of a chimaeric protein vaccine containing *Mycoplasma hyopneumoniae* antigens and LTB against experimental *M. hyopneumoniae* infection in pigs. Vaccine.

[33] Del Pozo SR, Thiry J, Vranckx K, López Rodríguez A, Chiers K, Haesebrouck F (2012). Efficacy of florfenicol injection in the treatment of Mycoplasma hyopneumoniae induced respiratory disease in pigs. Vet J.

[34] Vranckx K, Maes D, Marchioro SB, Villarreal I, Chiers K, Pasmans F (2012). Vaccination reduces macrophage infiltration in bronchus-associated lymphoid tissue in pigs infected with a highly virulent *Mycoplasma hyopneumoniae* strain. BMC Vet Res.

[35] Villarreal I, Maes D, Meyns T, Gebruers F, Calus D, Pasmans F (2009). Infection with a low virulent *Mycoplasma hyopneumoniae* isolate does not protect piglets against subsequent infection with a highly virulent *M. hyopneumoniae* isolate. Vaccine.

[36] Vicca J, Maes D, Jonker L, De Kruif A, Haesebrouck F (2005). Efficacy of in-feed medication with tylosin for the treatment and control of *Mycoplasma hyopneumoniae* infections. Vet Rec.

[37] Meyns T, Maes D, Dewulf J, Vicca J, Haesebrouck F, de Kruif A (2004). Quantification of the spread of *Mycoplasma hyopneumoniae* in nursery pigs using transmission experiments. Prev Vet Med.

[38] Meyns T, Maes D, Calus D, Ribbens S, Dewulf J, Chiers K (2007). Interactions of highly and low virulent *Mycoplasma hyopneumoniae* isolates with the respiratory tract of pigs. Vet Microbiol.

[39] Baskerville A (1972). Development of the early lesions in experimental enzootic pneumonia of pigs: an ultrastructural and histological study. Res Vet Sci.

[40] Sarradell J, Andrada M, Ramirez AS, Fernández A, Gómez-Villamandos JC, Jover A (2003). A morphologic and Immunohistochemical study of the bronchus-associated lymphoid tissue of pigs naturally infected with *Mycoplasma hyopneumoniae*. Vet Pathol.

[41] Nathues H, Spergser J, Rosengarten R, Kreienbrock L, Grosse Beilage E (2012). Value of the clinical examination in diagnosing enzootic pneumonia in fattening pigs. Vet J.

[42] Leon EA, Madec F, Taylor NM, Kobisch M (2001). Seroepidemiology of *Mycoplasma hyopneumoniae* in pigs from farrow-to-finisch farm. Vet Microbiol.

[43] Thacker EL, Thacker BJ, Kuhn M, Hawkins PA, Waters WR (2000). Evaluation of local and systemic immune responses induced by intramuscular injection of a *Mycoplasma hyopneumoniae* bacterin to pigs. Am J Vet Res.

[44] Woolley LK, Fell SA, Gonsalves JR JR, BBA R, Collins D, Kuit TA (2014). Evaluation of recombinant Mycoplasma hyopneumoniae P97/P102 paralogs formulated with selected adjuvants as vaccines against mycoplasmal pneumonia in pigs. Vaccine.

[45] Djordjevic SP, Eamens GJ, Romalis LF, Nicholls PJ, Taylor DJ, Chin J (1997). Serum and mucosal antibody responses and protection in pigs vaccinated against *Mycoplasma hyopneumoniae* with vaccines containing a denatured membrane antigen pool and adjuvant. Aust Vet J.

[46] Thacker EL, Thacker BJ, Young TF, Halbur PG (2000). Effect of vaccination on the potentiation of porcine reproductive and respiratory syndrome virus (PRRSV)-induced pneumonia by *Mycoplasma hyopneumoniae*. Vaccine.

[47] Thacker E, Strait EL, Ruebling K, Nilubol D, Erickson B, White R. Evaluation of organism number and the immune response to one dose *Mycoplasma hyopneumoniae* vaccines. In: Blaha T, Pahlitzsch C, editors. 18th IPVS Congress. Hamburg, Germany; 2000.

[48] Yang J, Hooper WC, Phillips DJ, Talkington DF (2004). Cytokines in Mycoplasma pneumoniae infection. Cytokine Growth Factor Rev.

[49] Van Reeth K, Van Gucht S, Pensaert M (2002). In vivo studies on cytokine involvement during acute viral respiratory disease of swine: troublesome but rewarding. Vet Immunol Immunopathol.

[50] Ahn KK, Kwon D, Jung K, Ha Y, Seo MJ, Kim S-H (2009). Identification of interleukin-1, tumor necrosis factor-α, and interleukin-6 expression in lungs from pigs naturally infected with *Mycoplasma hyopneumoniae* by in situ hybridization. J Vet Med Sci.

[51] Asai T, Okadaa M, Onoa M, Irisawaa T, Morib Y, Yokomizob Y (1993). Increased levels of tumor necrosis factor and interleukin 1 in bronchoalveolar lavage fluids from pigs infected with *Mycoplasma hyopneumoniae*. Vet Immunol Immunopathol.

[52] Rodriguez F, Ramireza GA, Sarradell J, Andradaa M, Lorenzo H (2004). Immunohistochemical Labelling of cytokines in lung lesions of pigs naturally infected with *Mycoplasma hyopneumoniae*. J Comp Pathol.

[53] Sibila M, Pieters M, Molitor T, Maes D, Haesebrouck F, Segalés J (2009). Current perspectives on the diagnosis and epidemiology of *Mycoplasma hyopneumoniae* infection. Vet J.

[54] Steenhard NR, Jungersen G, Kokotovic B, Beshah E, Dawson HD, Urban JFJ (2009). *Ascaris suum* infection negatively affects the response to a *Mycoplasma hyopneumoniae* vaccination and subsequent challenge infection in pigs. Vaccine.

[55] Stevenson GW, Done S, Thomson J, Varley M (1998). Bacterial pneumonia in swine. 15th IPVS congress.

[56] Martinod S, Moneti G, Vignola G (1996). Protection against *Mycoplasma hyopneumoniae* and *Actinobacillus pleuropneumoniae* infections using a mycoplasma inactivated vaccine Respisure under field conditions. 14th IPVS congress.

[57] Hodgins DC, Shewen PE, Dewey CE (2004). Influence of age and maternal antibodies on antibody responses of neonatal piglets vaccinated against *Mycoplasma hyopneumoniae*. J Swine Health Prod.

